# A retrospective study on the prevalence of main clinical findings in brown bears (*Ursus arctos*) rescued from substandard husbandry conditions

**DOI:** 10.3389/fvets.2023.1299029

**Published:** 2023-12-13

**Authors:** Elena Stagni, Sara Sequeira, Marta Brscic, Irene Redtenbacher, Sabine Hartmann

**Affiliations:** ^1^VIER PFOTEN International, Linke Wienzeile, Vienna, Austria; ^2^Department of Animal Medicine Production and Health (MAPS), University of Padova, Viale dell’Università, Legnaro PD, Italy

**Keywords:** brown bear, health, keeping condition, animal welfare, pre-rescue bear origin, clinical finding

## Abstract

Brown bears (*Ursus arctos*) are kept under varied captive conditions, some of which may greatly compromise their welfare. FOUR PAWS is an NGO that rescues some of these bears kept in substandard conditions and houses them in species-appropriate sanctuaries, where preventive and reactive veterinary care is provided. This retrospective study aims to provide an overview of pathologies and clinical abnormalities reported in veterinary records and their prevalence according to body system affected and pre-rescue bear origin. Origin was categorised as subzoo (bears coming from substandard zoos), dancing (used to “dance” upon a music cue), restaurant (used to attract clients), private keeping (used for various purposes, such as photo props), circus (used for shows), and bear-baiting (exploited for hunting dog training in baiting stations). Clinical findings were extracted from reports of veterinary examinations done from 2006 to 2021, during rescue, routinely, in response to clinical signs, and/or *post-mortem*. Their prevalence was calculated according to the body system affected and neoplasia (specific group independent from the organ) over the findings’ total number. Prevalence was also calculated according to pre-rescue origin (general and relative values in proportion to the number of reports per origin). Results refer to 302 veterinary reports of 114 bears examined, rescued from 1998 to 2021, with the age at rescue varying from a few months to 30 years (median 13 years). The total number of clinical findings was 1,003, and the systems with more findings were oral cavity (56.0%), abdominal cavity and digestive system (7.9%), integumentary (7.9%), ocular systems (7.7%), and musculoskeletal (7.6%). Findings involving other body systems and neoplasia were less prevalent (≤2.8%). Results showed a higher prevalence of some clinical findings for bears rescued from certain origins compared to others. Straightforward associations between pre-rescue origin and clinical findings were not feasible due to unknown anamnesis and details on pre-rescue conditions, and because some housing and management characteristics might be transversal to origins. Results suggest that bears rescued from certain origins were prone to specific clinical findings, supporting the need for the creation of *ad hoc* preventive veterinary and husbandry management plans after rescue, thus contributing to the improvement of captive bear welfare.

## 1 Introduction

Welfare is a multidisciplinary concept that includes, but is not limited to, physical health and emotional state, as described by Mellor et al. (1) in the Five Domains Model. In order to systematically and thoroughly assess animal welfare, this model identifies “health” as a domain that, together with “nutrition,” “physical environment,” and “behavioural interactions,” form the foundations for inferences regarding the fifth domain, the mental state (1). Preserving the health of animals should therefore contribute to a good welfare state by enabling the performance of natural and positive behaviours, and minimising negative experiences such as pain and discomfort.

Welfare is a subjective state and similar experiences, rearing and housing conditions may lead to different perceptions by individual animals (2, 3). Not all animals, indeed, display the same response or clinical signs when exposed to pathogens, stressors, painful conditions, and other suboptimal conditions, with some being more resilient than others. It has been suggested that bears are extremely resilient, being able to physically resist severe environmental conditions, and this may potentially lead to their neglect in captivity (4). Moreover, bears can reach a considerable advanced age in captivity (5), and like most captive animals, they live longer than their conspecifics in the wild (6–8). This advancing age is connected to the development of age-related health issues, some of which may be very painful (9). The onset and progression of some of these health issues can be influenced by an appropriate environment and management. The environment bears are kept in should be carefully designed and managed to meet their physical, emotional, social, and behavioural needs and should vary according to the season and specific physiological states of the animals (10). For example, bear nutrition should not only meet the nutritional requirements targeted for the specific physiologic state but should also fulfil the feeding ecology, nutritional strategies, and nutritional wisdom of the animal (11, 12). Thus, feed type, quality, and way of provision in captivity play an important role in bear health and welfare status (10, 13).

Unfortunately, captive bears may be kept in conditions that do not meet their requirements (14), such as brown bears (*Ursus arctos*) in substandard zoos, circuses, private keeping, baiting stations, or even used as dancing bears. These keeping conditions share the low to very low welfare standards in which animals are kept, from inappropriate physical environments to inadequate nutrition to even forcing postures and movements that are unnatural to bears. Specifically, dancing bears were forced to perform dancing-like movements upon the cue of a violin, whilst being chained by a metal ring on their nose and upper lip. These animals were trained from a very young age, being placed on a hot metal plate whilst a violin was being played, and the pain caused by the heat of the plate triggered a stomping motion reaction, perceived as “dancing.” The animal was thus conditioned, and the sound of the violin worked as the cue to “dance” (15). Bears in restaurants were used as props to attract clients and were usually kept in small, barren cages in front of the property. Both dancing and restaurant bears were often given alcohol (16, 17) in addition to an inadequate diet. Bears from private keeping were used for different purposes and kept in a variety of ways, from selfie bears that were chained by their nose ring and dragged around tourist locations so that people could take pictures with them (18) to bears kept as pets or in small enclosures as attractions in private properties or amusement parks. Bears kept in baiting stations were chained by their necks to a tree and used to train hunting dogs, whilst they spent the rest of their time in small and barren cages.

A gradual improvement of these bears’ welfare is achieved after their rescue and housing in species-appropriate sanctuaries, where specialised veterinary care is provided, and husbandry and the natural environment aim at stimulating species-specific behaviours (19, 20). Since 1998, the animal welfare organisation FOUR PAWS International (FP) has rescued many bears and housed them in species-appropriate sanctuaries. The health status of these animals is assessed through veterinary examinations performed both on a regular basis and when provision of specific medical care is needed.

This retrospective study aims to provide an overview of pathologies and clinical abnormalities reported in veterinary records and the prevalence of these clinical findings clustered according to body system affected and pre-rescue bear origin.

## 2 Materials and methods

### 2.1 Animals and veterinary reports

The data for the study were extracted from veterinary reports of medical examinations performed between 2006 and 2021 on brown bears housed in FP Sanctuaries in Europe. The reports have been compiled during pre-rescue and rescue veterinary examinations, routine health check-ups, examinations conducted in response to specific clinical signs reported by caretakers, specialist visits, and *post-mortem* examinations. The diagnostic tools used in each examination varied and included blood and urine analysis, radiology, ultrasonography, computed tomography, endoscopy, histopathology, cytology, and bacterial and fungal culture. Consultations with veterinary specialists, such as dentists, ophthalmologists, and cardiologists, were carried out on a necessity basis.

All information written in the veterinary reports were exported onto Microsoft Excel (version 2201), and they were related to: identity of the bear examined; year of rescue; bear age at rescue; pre-rescue origin categorised as subzoo (bears coming from substandard zoos), dancing (used to perform dancing-like movements upon a music cue), restaurant (used to attract clients), private keeping (used for different purposes, such as photo props), circus (used for shows), and bear-baiting (exploited for hunting dog training in baiting stations); date of examination; name of the veterinarian performing the examination; type of visit/diagnostic performed; and clinical findings. The experimental unit for the extracted data was the veterinary report. The frequency distribution of reports of veterinary examinations performed *in vivo* and *post-mortem* and their distribution through the years were calculated. The reports *in vivo* were further grouped into pre-rescue, rescue, and post-rescue. The frequency distribution of reports for each bear was calculated as such and according to pre-rescue origin. Finally, the frequency of the different types of visits/diagnostics was calculated based on the total number of reports.

### 2.2 Clinical findings

Each finding reported during the veterinary examination was considered an independent occurrence, even if the pathogenesis was correlated (e.g., *periapical abscess and open root*; *mucopurulent ocular discharge and conjunctivitis*). When the same finding on a bear was reported in more than one veterinary report over time, it was considered only once, unless the severity of the pathology changed (e.g., from *mild coxofemoral arthrosis* to *moderate coxofemoral arthrosis*) or the finding describing the pathology was at a different stage of progression (e.g., cavities and tooth destroyed). In case where a pathology concerned more than one anatomical part (e.g., two teeth), the finding (e.g., fracture) was considered once with the cumulative number of elements involved (e.g., fractures in two teeth).

In the second step, the prevalence of these clinical findings was calculated according to body system affected and neoplasia (allocated to a specific group, independently from the organs involved and counted only in the neoplasia group) as a percentage over the total number of findings. Laboratory results, such as bacteriological culture and histopathology, were included in the categories of body system affected and neoplasia. Parasitological examinations on faecal samples were not included in the dataset as feacal screenings are performed on a regular basis, independently from the veterinary examinations, and their results are managed in another data recording system. Each body system was subcategorised in order to group findings of a similar nature (e.g., *one or more missing teeth*; *lens luxation, uni- or bilateral cataract*). A further subgrouping was created within the subcategories for the oral and ophthalmic findings to reflect the variance found in the number of anatomical parts involved (e.g., *2 to 10 teeth missing*; *Bilateral cataract*). The groups of clinical findings and their descriptions are reported in Supplementary Table 1.

The prevalence of findings was also calculated according to pre-rescue origin (general and relative values in proportion to the number of reports for each origin).

## 3 Results

### 3.1 Animals and veterinary reports

**Figure 1:**
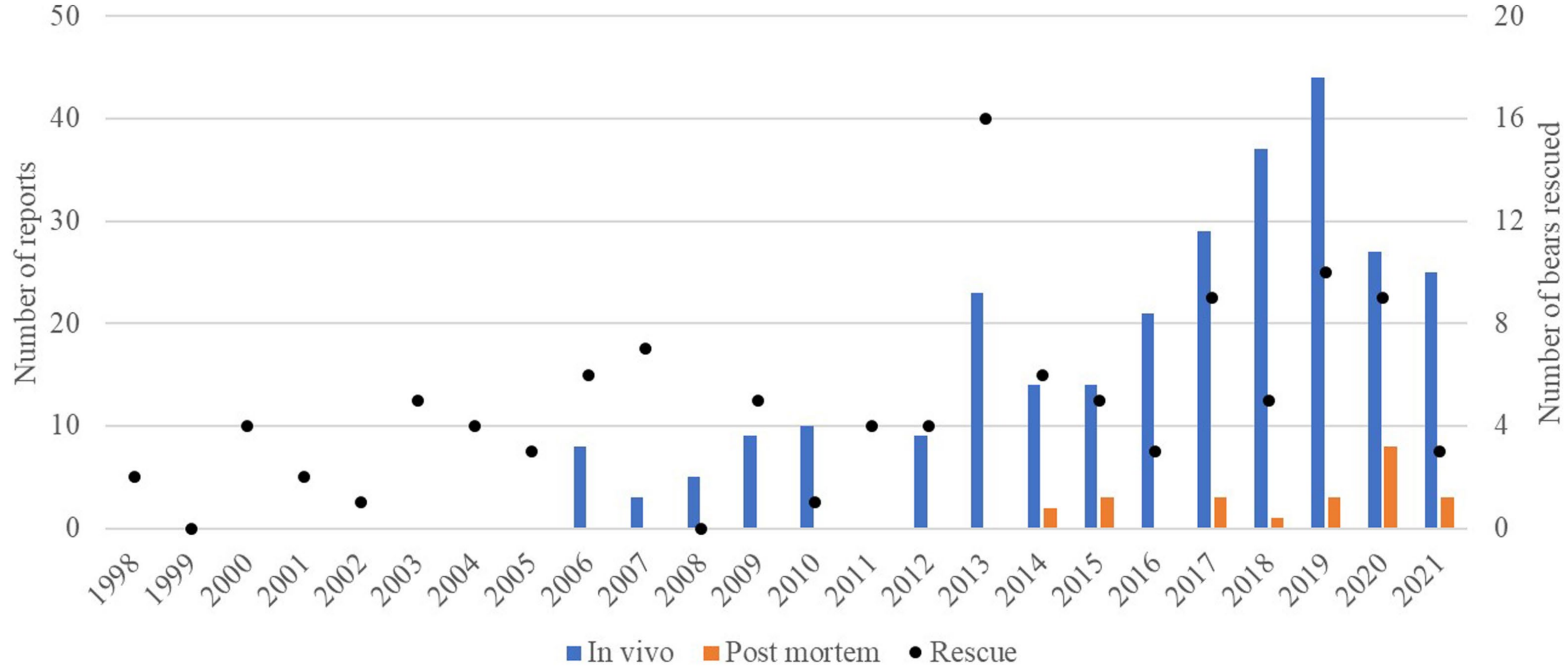
Distribution over years of the numbers of veterinary examination reports of brown bears (*Ursus arctos*) (no reports were collected before 2006): *in vivo* (blue) and *post-mortem* (orange) on the left axis scale. The points represent the number of bears rescued each year within the group of bears involved in this study (right axis scale).

**Figure 2:**
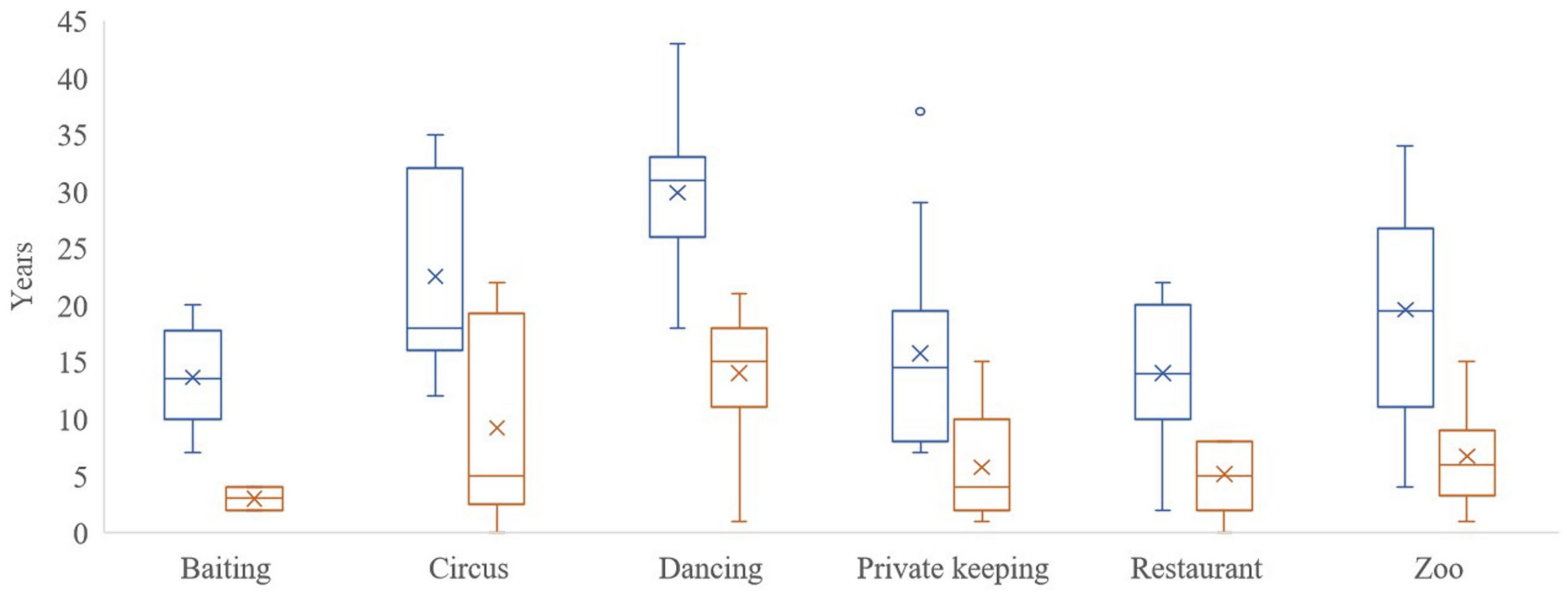
Box and whisker plot of the average age of the brown bears (*Ursus arctos*) (blue) and length of stay (orange) in FOUR PAWS sanctuaries until the end of 2021 or bear death, according to pre-rescue origin.

The results of this study refer to 302 veterinary reports of 114 brown bears inspected. The bears were housed in six FP brown bear sanctuaries: Bear Sanctuary Müritz in Germany (*n* = 24) (18) ear Sanctuary Arbesbach in Austria (*n* = 8) (18) ear Sanctuary Belitsa in Bulgaria (*n* = 33) (18) ear Sanctuary Domazhyr in Ukraine (*n* = 25) (18) ear Sanctuary Prishtina in Kosovo (*n* = 20), and Arosa Bear Sanctuary in Switzerland (*n* = 4). They were rescued from 1998 to 2021 (Figure 1), and the highest number of bear rescues per year was 16 in 2013, followed by 10 rescues in 2019 and nine rescues in 2017 and 2020. Bear age at rescue varied from a few months to 30 years old, with a median value of 13 years. They were rescued from the following origins: 32 were from subzoos, 27 were dancing bears, 23 were kept next to restaurants, 16 were in private custody, 10 were used in circuses, and 6 were used for bear-baiting. The average age of the bears and length of stay in the FP sanctuaries until the end of 2021 or bear death according to pre-rescue origin are shown in Figure 2.

The number of reports of veterinary examinations performed *in vivo* and *post-mortem* was 278 and 24, respectively. Within the *in vivo* reports, 14 (5.0%) were compiled during pre-rescue, 23 (8.3%) during rescue, and 241 (86.7%) during post-rescue veterinary examinations. The distribution of the number of reports through the years is also shown in Figure 1. The number of reports of *in vivo* examinations increased progressively from 2015 to 2019, with a peak of 44 during 2019. The highest number of *post-mortem* reports was registered in 2020 (*n* = 8). The number of reports per bear varied: 30 bears had one report, 26 had two reports, 26 had three reports, 23 had four reports, 4 had five reports, and 5 had six reports. Of the 30 bears with only one report, six of them had only a *post-mortem* report.

The frequency distribution of the reports according to pre-rescue origin showed that the majority of reports were from dancing (*n* = 90), followed by subzoos (*n* = 78), restaurants (*n* = 58), private keeping (*n* = 37), circuses (*n* = 25), and bear-baiting bears (*n* = 14). The 37 reports of pre-rescue and rescue examinations were performed on 30 bears, divided according to their origin as follows: nine restaurants, six bear-baiting, five circuses, five private keepings, four subzoos, and one dancing.

Within the medical examinations of these reports, the most frequent type of visit or diagnostics applied was the general clinical examination (G, *n* = 162), followed by blood analysis (18), dentist examination (D, *n* = 147), ultrasound (U, *n* = 109) and radiology (X, *n* = 84), in 45 different combinations, as shown in Supplementary Table 2.

### 3.2 Clinical findings

The total number of clinical findings extracted was 1,003, and the systems with more findings recorded in the reports were: oral cavity (56.0%), abdominal cavity and digestive system (7.9%), integumentary (7.9%), ocular (7.7%), and musculoskeletal (7.6%) systems. The other body systems and neoplasia showed an overall prevalence of findings ≤2.8%: neoplasia (2.8%), urinary system (2.6%), cardiovascular system (2.5%), respiratory system (1.4%), reproductive system (1.1%), haematopoietic and lymphatic system (0.9%), poor nutritional status (0.8%), neurological system (0.5%), and endocrine system (0.4%).

**Figure 3:**
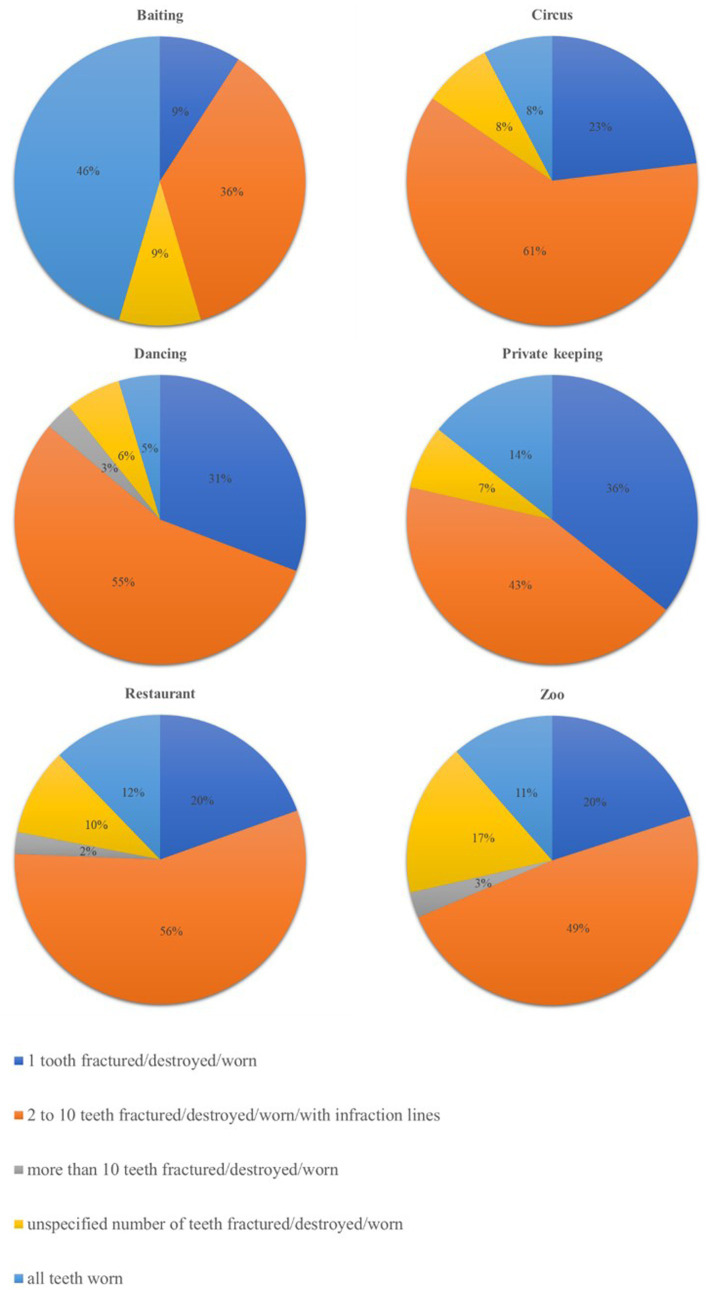
Distribution of the percentages of each subgroup of findings related to the subcategory of teeth fractured, destroyed, worn, or with attrition or infraction lines, according to the origin of the brown bears (*Ursus arctos*) in the dataset of the veterinary examination reports of the current study.

The raw number of findings related to the most represented body systems and neoplasia and the respective percentages, calculated on the total number of findings relative to the same system/neoplasia, are reported in Table 1. The prevalence of clinical findings according to the pre-rescue origin of the bears is also reported in Table 1, which showed that some findings tend to be reported more often in bears rescued from certain origins compared to others. For example, within the ocular system, findings related to the lens, such as luxation or uni−/bilateral cataracts, seemed more present in dancing bears compared to bears from other origins. Within the oral cavity system, the findings of teeth fractured, destroyed, worn, or with attrition seemed more represented in bears used for bear-baiting, dancing, and restaurant bears. These findings were the most numerous group in the oral cavity, and the percentage of the different subcategories for each origin is represented in Figure 3. Regarding neoplasia, the majority were malignant, and circus, subzoo, and dancing bears showed the apparently highest prevalence.

**Table 1:** Raw number of clinical findings related to the most represented body systems and neoplasia in the veterinary examination reports (*n* = 302), their respective percentages (calculated on the total number of findings relative to the same body system/neoplasia, sum up to 100%), and percentages of clinical findings according to the pre-rescue origin [calculated on the total number of reports per origin (*n* of reports)] of the brown bears (*Ursus arctos*) rescued and housed in FOUR PAWS Sanctuaries (*n* = 114).

Clinical finding	Number of findings (*n* = 1,003)	% on total findings per system	Origin					
			Bear-baiting (*n* = 14)	Circus (*n* = 25)	Dancing (*n* = 90)	Private keeping (*n* = 37)	Restaurant (*n* = 58)	Subzoo (*n* = 78)
*Oral cavity*								
One or more teeth affected by cavities	30	5.3	14.3	8.0	18.9	8.1	3.4	5.1
One or more teeth fractured, destroyed, worn or with attrition	179	31.9	78.6	52.0	72.2	37.8	70.7	44.9
One or more missing teeth	65	11.6	21.4	12.0	44.4	21.6	15.5	2.6
One or more teeth discoloured, demineralised and/or with an enamel defect	32	5.7	7.1	4.0	1.1	29.7	20.7	7.7
One or more open root	138	24.6	35.7	28.0	58.9	37.8	51.7	37.2
Gum, buccal mucosa, tongue lesions, perio, and/or endodontitis	55	9.8	14.3	16.0	16.7	27.0	19.0	16.7
Apical/periapical lesions, osteomyelitis, osteolysis, purulent infection, abscess and/or fistula	60	10.7	14.3	20.0	27.8	16.2	12.1	19.2
Malocclusion	1	0.2	7.1	0.0	0.0	0.0	0.0	0.0
Persistent milk tooth	2	0.4	0.0	0.0	0.0	2.7	0.0	1.3
*Abdominal cavity and digestive system*								
Rectal prolapse and paralysis	2	2.5	14.3	0.0	0.0	0.0	0.0	0.0
Peritonitis	3	3.8	0.0	0.0	2.2	0.0	0.0	1.3
Abdominal fluid, ascites, and hemoabdomen	7	8.9	0.0	8.0	3.3	0.0	0.0	2.6
Alteration of the gallbladder and biliary duct	36	45.6	7.1	32.0	0.0	0.0	17.2	21.8
Hepatic modifications and degeneration	12	15.2	0.0	16.0	3.3	2.7	1.7	3.8
Liver perivasculitis and secondary hepatitis	2	2.5	0.0	0.0	0.0	0.0	0.0	2.6
Esophagitis, gastritis and gastroduodenitis, gastrointestinal gas accumulation, stomach rupture	10	12.7	0.0	0.0	5.6	2.7	0.0	5.1
Pathologies of the small intestine	7	8.9	0.0	4.0	0.0	0.0	0.0	7.7
*Integumentary system*								
Alopecia, adnexal atrophy, and fur quality	12	15.2	0.0	4.0	7.8	5.4	3.4	0.0
Alteration of one or more pads/soles and of one or more claws	28	35.4	7.1	16.0	4.4	13.5	13.8	7.7
Hyperkeratosis, acanthosis, and hyperpigmentation	3	3.8	0.0	0.0	3.3	0.0	0.0	0.0
Dermatitis, pyodermatitis, and erythema	6	7.6	0.0	0.0	2.2	0.0	5.2	1.3
Cutaneous or subcutaneous nodules or masses, suspect of papilloma.	5	6.3	0.0	0.0	1.1	2.7	1.7	2.6
Abrasions and superficial ulcerations	2	2.5	0.0	0.0	1.1	0.0	0.0	1.3
Chronic or purulent abrasions, wounds, decubital lesions, and scars	23	29.1	7.1	4.0	3.3	21.6	5.2	9.0
*Ocular system*								
Signs of conjunctivitis and/or ocular discharge	8	10.4	0.0	0.0	1.1	5.4	8.6	0.0
Presence of *Thelazia* spp.	2	2.6	0.0	0.0	0.0	0.0	3.4	0.0
Foreign body in eye	1	1.3	0.0	0.0	0.0	0.0	1.7	0.0
Uni- or bilateral corneal hyperpigmentation, melanosis, and/or opacity	12	15.6	0.0	8.0	7.8	2.7	3.4	0.0
Uni- or bilateral corneal vascularization, edema, ulcer, and/or scar	4	5.2	0.0	0.0	1.1	8.1	0.0	0.0
Lens luxation, uni-, or bilateral cataract	25	32.5	0.0	4.0	21.1	5.4	1.7	2.6
Pathologies of uvea, sclera, and anterior chamber	8	10.4	0.0	0.0	6.7	2.7	0.0	1.3
Glaucoma	4	5.2	0.0	4.0	2.2	0.0	1.7	0.0
Unilateral retinal detachment or degeneration	7	9.1	0.0	4.0	6.7	0.0	0.0	0.0
Unilateral vitreous degeneration	1	1.3	0.0	0.0	1.1	0.0	0.0	0.0
Bilateral optical nerve degeneration	1	1.3	0.0	0.0	1.1	0.0	0.0	0.0
Unilateral phthisis bulbi	2	2.6	0.0	0.0	1.1	0.0	1.7	0.0
Unilateral microphakia	1	1.3	0.0	0.0	1.1	0.0	0.0	0.0
Unilateral microphthalmos	1	1.3	0.0	0.0	1.1	0.0	0.0	0.0
*Musculoskeletal apparatus*								
Alteration of the muscular tissue, muscle calcification, presence of radiodense material, and necrotic inflammation	6	7.9	7.1	4.0	1.1	2.7	0.0	2.6
Left or bilater femoropatellar/ femorotibial arthrosis or tarsal arthrosis	11	14.5	0.0	4.0	5.6	0.0	1.7	5.1
Spine arthrosis and/or spondylosis, degenerative spine changes, vertebral dislocation, tissue formation on the side of the vertebral body, dens axis chip	16	21.1	0.0	0.0	4.4	0.0	1.7	14.1
One or more discs herniated or protruded, degenerative discopathies	4	5.3	0.0	0.0	0.0	0.0	0.0	5.1
Uni- or bilateral coxofemoral osteoarthritis	17	22.4	0.0	0.0	6.7	8.1	1.7	9.0
Anatomical changes that caused modification of body structure and happened before the rescue	10	13.2	7.1	16.0	2.2	0.0	0.0	3.8
Uni- or bilateral elbow, carpal and metacarpal arthrosis, carpal sclerosis	9	11.8	0.0	4.0	1.1	0.0	1.7	7.7
Fracture, luxation or bone sclerotic changes	3	3.9	0.0	4.0	0.0	0.0	0.0	2.6
*Neoplasia*								
Benign neoplasia	7	25	7.1	0.0	1.1	2.7	0.0	5.1
Malignant neoplasia	21	75	0.0	12.0	10.0	2.7	0.0	10.3

## 4 Discussion

In this study, the prevalence of clinical findings reported in 302 veterinary records of examinations carried out on 114 brown bears was investigated across body systems. Results suggest that the most frequent findings in bears housed in FP sanctuaries rescued from substandard conditions concern the oral cavity, abdominal cavity and digestive system, integumentary system, ocular system, and musculoskeletal apparatus, in decreasing order. According to the results of a survey conducted by Blake and Collins (21) in 50 North American zoos and aquaria, integumentary (54 cases), gastrointestinal (45 cases), and ocular diseases (17 cases) represented the majority of the clinical findings of 512 bears. The direct comparison of the distributions of findings across body systems between the current study and that by Blake and Collins (21) should be done with caution considering their different natures (direct data vs. survey data), study population, and statistical unit. In fact, the survey carried out by Blake and Collins (21) includes responses regarding bears in different rearing conditions and belonging to seven *Ursidae* species. The same direct comparison limitation applies to the results of most relevant diseases described in other retrospective studies, differing from the current one in that they were applied to subpopulations of captive bears of different species and ages, with a focus on specific anatomical systems, and/or assessed only *post-mortem* (22–25). For example, Clark et al. (26) focused on dental and temporomandibular joint pathologies, and Kitchener and MacDonald (27) focused on skeletal and dental pathologies. A few studies described the most common diseases developed in geriatric animals (4, 5, 27). Several others were based on data collected from autopsy results or skeleton examinations (23, 25, 26, 28, 29). To the best of the authors’ knowledge, the current study represents the retrospective assessment of the largest collection of veterinary reports of captive brown bears across ages, including juvenile, adult, and geriatric animals that were examined *in vivo* and/or *post-mortem*.

The number of reports increased gradually throughout the years, with the exception of a spike of 23 reports in 2013, concomitant with a high number of rescues. This gradual increment of reports reflects the improvement of the record-keeping system in the organisation, the higher number of bears housed in the sanctuaries, and their ageing, which predisposes them to more frequent medical attention. The highest number of reports was recorded in 2019, when FP adopted the managerial choice of conducting as many routine veterinary examinations as possible, thus striving to preventively reduce the need for curative interventions.

As expected, bears were mainly examined post-rescue, and the limited number of pre- and during-rescue reports was due to restricted access to the bears during previous management. Moreover, poor examination conditions (e.g., no lighting) and limited time and resources (e.g., diagnostic tools) might have underestimated clinical findings, leading the authors to hypothesise that post-rescue findings reported by the veterinarians might have included health issues that were already present upon rescue. Other health issues might have developed after rescue and over time because of natural ageing processes or isolated events, such as accidents. The distinction between the period (pre- or post-rescue) of the onset of the health issue was, indeed, not the aim of the current study.

The system with the highest number of findings was the oral cavity, which is commonly affected in bears in captivity, as reported by Bourne et al. (9). Most of these findings were part of the category of teeth fractured, destroyed, worn, or with attrition, followed by open roots. Accordingly, Kitchener (4) described that the most common problem diagnosed on the skulls of captive bears was broken or open-tipped canines (>70%). This type of problem, together with wear and enamel erosion, can be attributed to compulsive chewing of the cage bars, a stereotypical behaviour developed in an inadequate environment (4, 9, 10). This bar-biting behaviour might also explain the high prevalence of teeth fractured, destroyed, worn, or with attrition reported for bears rescued from baiting stations, where they are usually kept in small barren cages when not used to train hunting dogs.

Dancing bears seem to be generally more at risk of developing health issues in the oral cavity than bears of other origins. The highest percentage of reported missing teeth might be associated with dietary and feeding aspects since an inappropriate diet has a role in developing medical issues of the oral cavity, although this managerial condition is reportedly common across origins. Moreover, missing teeth might be explained, on the one hand, by anecdotes reporting that previous owners of dancing bears extracted bears’ teeth for their own personal safety, but no scientific evidence has been gathered of such a practice, making it a rather speculative statement. On the other hand, dancing bears have been under FP care for longer than other origins, and teeth might have been extracted due to their bad state during repeated veterinary interventions. In fact, dancing bears showed the highest percentage of cavities, open roots, and apical/periapical lesions, osteomyelitis, osteolysis, purulent infection, abscess, and/or fistula.

Regarding the abdominal cavity and digestive system, the highest prevalence of alterations of the gallbladder and biliary duct, hepatic modifications, and degeneration was reported for circus bears. According to Blake and Collins (21), hepatobiliary issues are not uncommon in bears in captivity. However, it is difficult to relate this finding to the circus origin, considering that alcohol and/or an inappropriate and unbalanced diet, which act as possible predisposing factors for liver degeneration (13), are traits that could be found across different pre-rescue origins. Gastroenteric and hepatobiliary systems are also commonly affected by neoplasia (10, 21), and in the current study, they represent the majority of malignant neoplasia found in bears housed by FP (11/21), although they have been considered altogether with neoplasia of other body systems following the example of Föllmi et al. (5).

Bears in captivity also frequently suffer from skin conditions, especially hair loss and rough hair coats, typically diagnosed in polar (*Ursus maritimus*) (30, 31) and Andean (*Tremarctos ornatus*) bears (32, 33). In fact, the brown bears included in this study were more frequently affected by alterations of pads/soles and claws, and chronic and purulent abrasions, wounds, decubital lesions, and scars than by alopecia and decreased fur quality. According to Collins (10), warm temperatures, a constantly moist environment, abrasive and hard substrates, and inappropriate surface cleanliness are to predispose factors for such findings. Moreover, the majority of the bears performed some type of stereotypical behaviour, typically pacing and circling or figure eight walking (34), which predisposes animals to sole issues when performed on abrasive substrates. Circus bears, along with those rescued from restaurants and private keeping, seem subject to the alteration of pads/soles and claws, likely sharing similar predisposing housing conditions. Private keeping, although varying in terms of housing and management, seems predisposing to a higher percentage of chronic and purulent abrasions, wounds, decubital lesions, and scars compared to other origins.

Ophthalmic pathologies are not commonly described in bears, except for some case studies (35–37), an epidemiological study on brown bears (38), and a survey on giant pandas (*Ailuropoda melanoleuca*) (39). In the current study, lens conditions, such as luxation and uni- or bilateral cataract, were the most commonly reported findings. The majority of ophthalmic alterations were found in reports of dancing bears, which could be expected to be the oldest group of bears in this study. It could also be related to the suggestion by Stades et al. (40) that the use of a stick on the face of dancing bears could be the cause of the lens luxation and retinal detachment.

Degenerative joint disease and consequent mobility problems represent a painful syndrome that develops over a number of years and might be caused by the ageing process, inflammation, or infection (9). Osteoarthritic changes have been found in all the bears radiographed by Föllmi (41); therefore, it is not surprising that the musculoskeletal system was one of the more represented systems in this study, with the greatest number of findings related to uni- or bilateral coxofemoral osteoarthritis. Kitchener (4) detected osteophytes in the sacral area and hip joint in more than 75% of the bear skeletons studied. In the current study, reports with findings related to this group were from bears rescued from subzoos, private keeping, and dancing, in decreasing order. Subzoo bears also showed the highest number of cases of spine arthrosis and/or spondylosis, degenerative spine changes, vertebral dislocation, tissue formation on the side of the vertebral body, and dens axis chip, which was the second most frequent group of findings. In fact, according to Nunn et al. (42), bears showed the highest prevalence of spondyloarthropathy among carnivores (27%), and it was detected in 96% of the skeletons examined by Kitchener (4). Obesity, a predisposing factor for osteoarthritis, has been described as a problem in several zoo animals, including bears (9). However, bears rescued from substandard zoos were rather underweight; therefore, obesity might not be the explanation for their observed higher prevalence in this study. In general, rescued bears were reported to show low body condition scores and signs of malnutrition, rather than being overweight. The importance of an adequate diet and physical exercise that guarantee a correct body mass according to the bear’s physiological status has been reported to contribute to the prevention of joint problem development (9, 41). It is likely that these aspects were neglected in the previous keeping of these bears, regardless of their origin. Moreover, circus bears showed a higher prevalence of anatomical changes that caused body structure modifications, such as amputation or ankylosis, that were already present at rescue.

Inferential statistics from the results of this study on the brown bear population in captivity are limited due to the specificity of this study (convenience sample of bears rescued by FP and housed in FP sanctuaries). Moreover, the lack of precise information did not allow a straightforward association between pre-rescue bear origin and clinical findings. Particularly because of the unknown anamnesis and the difficulty to ascertain details on housing, environment, management, and handling of these bears prior to rescue. In addition, some characteristics, such as improper nutrition, a lack of enrichment, or species-appropriate stimuli, were likely transversal to all pre-rescue origins.

Data were presented as the prevalence of findings according to pre-rescue origin, although a limitation of this study is represented by the distribution of the number of veterinary reports among origins. Indeed, the majority of the reports belonged to dancing and subzoo bears, which could be explained by the fact that they were rescued in larger numbers and in earlier times. In the same way, it is not surprising that bears rescued from baiting stations were the least represented, as this practice became illegal only recently in Ukraine and is still practised in Russia. Thus, only a few of these bears have been somewhat recently rescued from illegal baiting or fighting stations in Ukraine. Furthermore, it could be argued that the number of veterinarians performing the examinations could cause a lack of standardisation, due to individual differences as well as different reporting methods (e.g., during dental examinations, different veterinarians may report cavity severity differently). In fact, human perception influences disease detection (43), and multiple people might assess the same conditions differently (44); therefore, the quality of the data should be considered before any quantitative analysis and interpretation (45). In favour of veterinarians performing examinations on bears housed by FP, it could be pointed out that they all had the same purpose of guaranteeing the maximum possible health and welfare state for each single animal, and this might have consequently reduced the possible bias due to individual perception. Moreover, the reports were collected by a centralised office, making sure the same kind of data was provided.

In conclusion, this retrospective study carried out on veterinary examination records provided an overview of the prevalence of disease and health issues in rescued brown bears in FP sanctuaries. The investigation of the conditions of captive wild animals, which are far less studied compared to those of farm and companion animals, is important to gather knowledge and provide evidence for decision-making at both practical and theoretical levels. The higher prevalence of some medical findings for bears rescued from certain pre-rescue origins may be used for the creation of *ad hoc* preventive veterinary and husbandry management plans, thus contributing to the improvement of captive brown bear welfare. Such plans should also consider the impact that each medical condition has on animal welfare, in terms of how pain, discomfort, or affected behaviours directly influence the mental state of the animal. The investigation of the role of specific husbandry and managerial aspects as possible preeminent predisposing factors, along with the strategies to overcome them, would be interesting topics for further studies.

## Data availability statement

The datasets presented in this article are not readily available because the dataset can be requested to VIER PFOTEN International. Requests to access the datasets should be directed to Elena.Stagni@vier-pfoten.org.

## Ethics statement

Ethical review and approval was not required for the study with animals in accordance with the local legislation and institutional requirements. Written informed consent from the owners of the animals was not required to participate in this study in accordance with the national legislation and the institutional requirements.

## Author contributions

ES: Conceptualization, Data curation, Investigation, Methodology, Project administration, Visualization, Writing – original draft, Writing – review & editing. SS: Data curation, Methodology, Writing – original draft, Writing – review & editing. MB: Conceptualization, Data curation, Methodology, Visualization, Writing – original draft, Writing – review & editing, Investigation. IR: Supervision, Writing – review & editing. SH: Funding acquisition, Supervision, Writing – review & editing.
