# Supplementary material for: A retrospective study on the prevalence of main clinical findings in brown bears (Ursus arctos) rescued from substandard husbandry conditions
Source: Front Vet Sci. 2023 Dec 13;10:1299029. doi: 10.3389/fvets.2023.1299029 (PMC10773888; doi:10.3389/fvets.2023.1299029)
Supplement: Supplementary file 1 [file Table_1.docx]

Supplementary Table 1 – Clinical findings recorded in veterinary examination reports (n=302) of brown bears *(Ursus arctos)* rescued from substandard husbandry conditions and housed in FOUR PAWS Sanctuaries, grouped per body system and neoplasia and in subcategories of similar nature.

| **Body system and neoplasia** |  | **Category and subcategory** | **Description** |
| --- | --- | --- | --- |
| 1. Oral cavity | **1.1** | **One or more teeth affected by cavities** | one or more teeth with a cavity, or one or more cavities on a tooth |
|  | **1.2** | **One or more teeth fractured, destroyed, worn or with attrition** | one or more teeth fractured, destroyed, worn, with attrition or with infraction lines |
|  | *1.2.1* | *1 tooth fractured, destroyed, worn or with attrition* | |
|  | *1.2.2* | *2-10 teeth fractured, destroyed, worn, with attrition or with infraction lines* | |
|  | *1.2.3* | *more than 10 teeth fractured, destroyed, worn or with attrition* | |
|  | *1.2.4* | *unspecified number of teeth worn or with attrition* | |
|  | *1.2.5* | *all teeth worn* | |
|  | **1.3** | **One or more missing teeth** | one or more missing teeth |
|  | *1.3.1* | *1 tooth missing* | |
|  | *1.3.2* | *2-10 teeth missing* | |
|  | *1.3.3* | *11-20 teeth missing* | |
|  | *1.3.4* | *more than 20 teeth missing* | |
|  | **1.4** | **One or more teeth discoloured, demineralised and/or with enamel defect** | one or more teeth discoloured, yellow, with enamel defect and black morsal discolouration |
|  | *1.4.1* | *1 tooth discoloured, pink or with enamel defect* | |
|  | *1.4.2* | *2-10 teeth discoloured, demineralised or with enamel defect* | |
|  | *1.4.3* | *more than 10 teeth discoloured or with enamel defect* | |
|  | *1.4.4* | *unspecified number of teeth discoloured, yellow, with enamel defect and black morsal discolouration* | |
|  | **1.5** | **One or more open root** | one or more teeth with open roots regardless of the fact that the tooth has been extracted |
|  | *1.5.1* | *1 tooth with open root* | |
|  | *1.5.2* | *2-10 teeth with open roots* | |
|  | *1.5.3* | *more than 10 teeth with open roots* | |
|  | *1.5.4* | *unspecified number of teeth with open roots* | |
|  | **1.6** | **Gum, buccal mucosa, tongue lesions, perio and/or endodontitis** | gingivitis, gingival overgrowth, gingival wound, lip mucosa mass, tongue scar, part of tongue missing, periodontic lesion, periodontitis, perio-endo and endodontitis |
|  | *1.6.1* | *tongue scar or missing parts* | |
|  | *1.6.2* | *gingival overgrowth or wound, mass lip mucosa, gingivitis, periodontic lesions* | |
|  | *1.6.3* | *perio-endo and endodontic lesions* | |
|  | **1.7** | **Apical/periapical lesions, osteomyelitis, osteolysis, purulent infection, abscess, fistula** | one or more teeth with periapical process or osteomyelitis or osteolysis or purulent infection or abscess, apical lesion or osteolysis, or fistula |
|  | *1.7.1* | *1 tooth with periapical process or osteomyelitis or purulent infection or abscess, apical lesion or osteolysis, or fistula* | |
|  | *1.7.2* | *2-4 teeth with periapical lesion or osteomyelitis or osteolysis, or fistula, apical lesion* | |
|  | *1.7.3* | *unspecified number of teeth with periapical osteomyelitis or osteolysis* | |
|  | **1.8** | **Malocclusion** | malocclusion |
|  | **1.9** | **Persistent milk tooth** | presence of one milk tooth |
| 2. Ocular system | **2.1** | **Signs of conjunctivitis and/or ocular discharge** | conjunctivitis in one eye, ocular discharge or mucopurulent discharge from one or both eyes |
|  | **2.2** | **Presence of *Thelazia spp.*** | presence of *Thelazia spp* in one eye |
|  | **2.3** | **Foreign body in eye** | presence of a foreign body (cornstalk) in one eye |
|  | **2.4** | **Uni- or bilateral corneal hyperpigmentation, melanosis and/or opacity** | corneal hyperpigmentation or opacity in one or both eyes, corneal melanosis |
|  | *2.4.1* | *Unilateral corneal (hyper-)pigmentation, melanosis or opacity* | |
|  | *2.4.2* | *Bilateral corneal hyperpigmentation or opacity* | |
|  | **2.5** | **Uni- or bilateral corneal vascularization, edema, ulcer and/or scar** | uni- or bilateral corneal vascularisation, edema, ulcer or scar |
|  | *2.5.1* | *Unilateral corneal vascularisation* | |
|  | *2.5.2* | *Bilateral corneal edema, ulcer or scar* | |
|  | **2.6** | **Lens luxation, uni- or bilateral cataract** | lens luxation in one eye, uni- or bilateral cataract |
|  | *2.6.1* | *Lens luxation* | |
|  | *2.6.2* | *Unilateral cataract* | |
|  | *2.6.3* | *Bilateral cataract* | |
|  | **2.7** | **Pathologies of uvea, sclera and anterior chamber** | uni- or bilateral pathology or lesion affecting the uvea, the sclera or the anterior chamber |
|  | *2.7.1* | *Unilateral uveitis, scleritis, cystic lesions on the iris, anterior chamber opacity or fibrin precipitation* | |
|  | *2.7.2* | *Bilateral synechia* | |
|  | **2.8** | **Glaucoma** | uni- or bilateral high intraocular pression or glaucoma |
|  | *2.8.1* | *Unilateral IOP or glaucoma* | |
|  | *2.8.2* | *Bilateral IOP or glaucoma* | |
|  | **2.9** | **Unilateral retinal detachment or degeneration** | unilateral retinal detachment or degeneration |
|  | **2.10** | **Unilateral vitreous degeneration** | unilateral vitreous degeneration |
|  | **2.11** | **Bilateral optical nerve degeneration** | bilateral optical nerve degeneration |
|  | **2.12** | **Unilateral phthisis bulbi** | unilateral phthisis bulbi |
|  | **2.13** | **Unilateral microphakia** | unilateral microphakia |
|  | **2.14** | **Unilateral microphthalmos** | unilateral microphthalmos |
| 3. Neoplasia | **3.1** | **Benign neoplasia** | hamartoma, gingival fibroma, dermatofibroma, duodenal adenoma, mammary adenoma, , splenic myelolipoma and gastric leiomyoma |
|  | **3.2** | **Malignant neoplasia** | mammary tubuloacinar carcinoma, mammary adenoma-adenocarcinoma, pancreatic tubular adenocarcinoma, pancreatic metastasis, bile duct carcinoma, bile duct tumour, hepatic neoplasia, malignant endocrine tumour in the liver, splenic lymphoma, stomach neoplasia, jejunal neoplasia, jejunal polypoid adenocarcinoma, adrenal tumour, mesentery melanoma, melanoma on the lip, melanoma in the pelvic limb, cutaneous sarcoma in the inguinal area, metastasis. |
| 4. Respiratory System | **4.1** | **Rhinitis and uni- or bilateral nasal discharge** | catarrhal purulent rhinitis, chronic nasal discharge, presence of *Pseudomonas chlororaphis* in the nose cavity and unilateral mucopurulent discharge |
|  | **4.2** | **Trachea and tracheobronchial lymph nodes** | tracheomalacia and size increase of the tracheobronchial lymph nodes |
|  | **4.3** | **Hemothorax** | hemothorax |
|  | **4.4** | **Deposit and metaplasia in lungs** | anthracosis and benign disseminated pulmonary osseous metaplasia |
|  | **4.5** | **Pulmonary edema** | Pulmonary edema or pulmonary cardiac changes |
|  | **4.6** | **Pneumonia** | pneumonia or purulent pneumonia |
| 5. Urinary System | **5.1** | **Uni- or bilateral modification of the renal structure and function** | nephritis, renal degenerative changes, pyelitis, amyloidosis, unclear corticomedullar junction, chronic renal disease, unilateral kidney hyperechogenic areas |
|  | **5.2** |  |  |
|  | **5.3** | **Bladder content or stenosis** | bladder sediment, hyperechogenic content or stenosis |
|  | **5.4** | **Alterations of the urine** | hematuria, isosthenuria, proteinuria |
| 6. Reproductive System | **6.1** | **Affection of the mammary apparatus** | pseudopregnancy, unilateral mammary swelling and presence of *Staph. schleiferi* in mammary gland secretion |
|  | **6.2** | **Pathologies of the ovary and glands penis** | ovarian cyst and balanitis |
| 7. Integumentary System | **7.1** | **Alopecia, adnexal atrophy and fur quality** | alopecia in specific areas or symmetrical and/or multifocal, adnexal glands atrophy and reduced fur quality |
|  | **7.2** | **Alteration of one or more pads/soles and of one or more claws** | sole cracks, sole discolouration or with ischemic-hyperemic areas, pad depigmentation, softening, callus, erythema, wound, two toe pad fused, presence of radiodense metal particles, claws infected, overgrown and bent, worn, broken |
|  | **7.3** | **Hyperkeratosis, acanthosis and hyperpigmentation** | hyperkeratosis, acanthosis and abdominal hyperpigmentation |
|  | **7.4** | **Dermatitis, pyodermatitis and erythema** | exudative or interdigital dermatitis, pyodermatitis, dermal lymphocytes-granulocytes infiltration, erythema |
|  | **7.5** | **Cutaneous or subcutaneous nodules or masses suspect of papilloma.** | cutaneous or subcutaneous nodules or mass on the abdomen, suspect of papilloma on the lip |
|  | **7.6** | **Abrasions or superficial ulcerations** | abrasions, superficial skin ulcerations. |
|  | **7.7** | **Chronic or purulent abrasions, wounds, decubital lesions and scars** | chronic abrasions, purulent abrasions, infected and/or contused or bite wounds, decubital lesions, scars |
| 8. Neurological System | **8.1** | **Degenerative neurological diseases** | multifocal lipofuscinosis, chronic degenerative myelopathy of Waller or neurogenic disorder |
|  | **8.2** | **Edema, trauma** | suspected traumatic neuropathy of the left peroneal branch of the lumbosacral plexus, edema, compression on the medulla oblongata |
| 9. Poor Nutritional State | **9.1** | **Low BCS / malnutrition** | a poor body condition score and/or malnutrition |
| 10. Endocrine System | **10.1** | **Uni- or bilateral thyroid cyst** | uni- or bilateral thyroid cyst |
| 11. Abdominal Cavity and Digestive System | **11.1** | **Rectal prolapse and paralysis** | anal prolapse and paralysis of the anal sphincter |
|  | **11.2** | **Peritonitis** | fibrinous or haemorrhagic and/or purulent peritonitis |
|  | **11.3** | **Abdominal fluid, ascites and hemoabdomen** | free abdominal fluid, ascites or hemoabdomen |
|  | **11.4** | **Alteration of the gallbladder and biliary duct** | biliary cirrhosis, bile duct thickening or hyperechogenic or with suspected fibrosis, gallbladder enlarged, with cholelith, with hyperechogenic areas or walls, with suspected fibrosis |
|  | **11.5** | **Hepatic modifications and degeneration** | liver hyperechogenic areas or lesions, multifocal white spot, suspected fibrosis and fibrotic areas, degenerative changes, nodular lesions or acute liver congestion |
|  | **11.6** | **Liver perivasculitis and secondary hepatitis** | liver perivasculitis and secondary purulent hepatitis |
|  |  |  |  |
|  | **11.7** | **Esophagitis, gastritis and gastroduodenitis, gastrointestinal gas accumulation, stomach rupture** | gastrointestinal gas accumulation, erosive-ulcerative esophagitis, erosive or erosive-ulcerative gastritis, haemorrhagic gastroduodenitis, stomach rupture |
|  | **11.8** | **Pathologies of small intestine** | thickening of small intestine mucosa, small intestine constriction, pylorus-duodenum stenosis, intussusception, polypoid mucosal hyperplasia, necrotic enteritis |
| 12. Haematopoietic and Lymphatic System | **12.1** | **Secondary lymphadenitis, splenitis** | secondary purulent lymphadenitis or splenitis |
|  | **12.2** | **Alteration of the size and appearance of the spleen** | splenic hyperplasia, splenomegaly, hyperechogenic areas, suspected fibrotic areas or acute splenic congestion |
|  |  |  |  |
|  | **12.3** | **Splenic gangrene** | splenic gangrene |
| 13. Cardiovascular System | **13.1** | **Heart insufficiency** | chronic heart insufficiency, left chronic heart insufficiency, left heart hypertrophy, radiological cardiac changes, systolic heart murmur, systolic cardiac dysfunction, insufficiency of all or mitral or pulmonary cardiac valve, aortic valve hyperechogenic changes, right heart enlarged and right ventricle dilatation, dilated cardiomyopathy, left ventricle papillary muscle hypertrophy |
|  | **13.2** | **Hematomas** | hematomas on the tongue |
|  | **13.3** | **Endocarditis and hydropericardium** | endocarditis and hydropericardium |
| 14. Musculoskeletal Apparatus | **14.1** | **Alteration of the muscular tissue, muscle calcification, presence of radiodense material and necrotic inflammation** | pelvic or hind quarter muscular atrophy, calcification, presence of radiodense material in the masseter or purulent necrotic inflammation |
|  | **14.2** | **Left or bilateral femoropatellar/femorotibial arthrosis or tarsal arthrosis** | left or bilateral femoropatellar/femorotibial arthrosis or tarsal arthrosis |
|  | **14.3** | **Spine arthrosis and/or spondylosis, degenerative spine changes, vertebral dislocation, tissue formation on the side of vertebral body, dens axis chip** | spine arthrosis and/or spondylosis, lumbar arthrosis and spondylosis, degenerative spine changes, C1/C2 dislocation, tissue formation on the side of vertebral body, dens axis chip |
|  | **14.4** | **One or more discs herniated or protruded, degenerative discopathies** | herniated disc or discs, discs protrusion, degenerative discopathies |
|  | **14.5** | **Uni- or bilateral coxofemoral osteoarthritis** | uni- or bilateral coxofemoral osteoarthritis |
|  | **14.6** | **Anatomical changes that caused modification of body structure and happened before the rescue.** | unilateral forearm underdevelopment, missing or curved radius, chronic elbow dislocation, distorted digitus, fingers dislocated, phalanges missing, tail avulsion |
|  | **14.7** | **Uni- or bilateral elbow, carpal and metacarpal arthrosis, carpal sclerosis** | uni- or bilateral elbow arthrosis, humeroulnar osteoarthritis, radial styloid process arthrosis, carpal sclerosis or osteoarthrosis, metacarpocarpal arthrosis |
|  | **14.8** | **Fracture, luxation or bone sclerotic changes** | fracture of rostral mandibular symphysis, unilateral right radius luxation, sclerotic changes in left tibial plateau |
