# Supplementary material for: A retrospective study on the prevalence of main clinical findings in brown bears (Ursus arctos) rescued from substandard husbandry conditions
Source: Front Vet Sci. 2023 Dec 13;10:1299029. doi: 10.3389/fvets.2023.1299029 (PMC10773888; doi:10.3389/fvets.2023.1299029)
Supplement: Supplementary file 2 [file Table_2.docx]

Supplementary Table 2. Combinations of type of visit/diagnostic performed during the veterinary examinations of brown bears *(Ursus arctos)* rescued from substandard husbandry conditions and housed in FOUR PAWS Sanctuaries, and number of reports in which the combination was present: G = General clinical exam, B = Blood analysis, X = radiology, U = ultrasonography, CT = computed tomography, FD = further diagnostic (endoscopy, urine analysis, histopathology, cytology and bacterial or fungal culture), D = dental specialistic examination, O = ophthalmological specialistic examination, Ca = cardiological specialistic examination, PM = post-mortem.

| Type of visit/diagnostic combination | Number of reports |
| --- | --- |
| G | 12 |
| B | 8 |
| G + B | 25 |
| G + U | 2 |
| G + FD | 1 |
| G + CT | 2 |
| G + B + U | 13 |
| G + B + X | 15 |
| G + B + U + X | 24 |
| G + U + X | 2 |
| G + B + X + FD | 2 |
| G + B + CT | 1 |
| G + B + FD | 3 |
| G + B + U + FD | 6 |
| G + B + U + X + FD | 6 |
| G + B + U + CT | 2 |
| G + B + CT + FD | 1 |
| D | 92 |
| D + B | 3 |
| D + X | 1 |
| D + B + X | 1 |
| D + U + X | 1 |
| D + B + U + X | 4 |
| D + B + U + FD | 1 |
| G + D | 2 |
| G + D + B | 3 |
| G + D + B + U | 13 |
| G + D + B + X | 1 |
| G + D + U + X | 4 |
| G + D + U + FD | 2 |
| G + D + B + U + X | 13 |
| G + D + B + U + FD | 3 |
| G + D + B + O + U | 1 |
| O | 1 |
| D + O + U | 1 |
| B + U + FD | 1 |
| G + D + O + U | 1 |
| G + Ca + U | 1 |
| G + D + Ca + U | 1 |
| FD | 2 |
| PM | 10 |
| PM + CT | 3 |
| PM + FD | 7 |
| PM + X | 3 |
| PM + B + U + CT | 1 |
